# Clinical characteristics and analysis of cerebrospinal fluid biochemical, tumor markers and cytologic indices in 209 patients with meningeal carcinomatosis: a cross-sectional study

**DOI:** 10.3389/fonc.2025.1594662

**Published:** 2025-10-09

**Authors:** Shaoqiang Xu, Chunxia Huang, Yuanyang Ye, Keyuan Lai, Sihan Lan, Jinhao Chen

**Affiliations:** Department of Laboratory, Guangdong Sanjiu Brain Hospital, Guangzhou, China

**Keywords:** meningeal carcinomatosis, clinical features, cerebrospinal fluid cytology, cerebrospinal fluid tumor markers, cross-sectional study

## Abstract

**Objective:**

To analyze the clinical characteristics and cerebrospinal fluid (CSF) biochemical indexes, tumor markers (TM) and cytomorphological indexes of patients with meningeal carcinomatosis (MC), and to explore the clinical application value of the above indexes.

**Methods:**

Retrospectively included 209 patients with MC in 2021–2023 into the study and collected their clinical information and laboratory data, compared the differences in indicators and analyzed the cytomorphological features and dynamic changes.

**Results:**

(i) The primary foci of MC patients were mostly lung cancer (86.6%), and the clinical manifestations did not change according to the tumor of the primary foci. The main manifestations were headache (65.55%) and meningeal enhancement (67.20%); (ii)Most MC patients (96.17%) had abnormal CSF biochemical indices, with some variations among primary foci; (iii) CYFRA21–1 level in CSF of MC patients was significantly higher than that in serum (P<0.05); (iv) The first detection rate of atypical cells in CSF of MC patients in our center was 95.22%, and the morphological characteristics were correlated with the primary foci; (v)Dynamic monitoring showed that the level of TM in CSF was consistent with the changes in the proportion of atypical cells in CSF, which could suggest fluctuations in the disease.

**Conclusion:**

In this study, it is clear that the clinical characteristics of MC patients and the CSF indicators have specific distribution patterns, and the combined analysis of CSF biochemical indicators, TMr levels and cytomorphology can provide a key basis for the diagnosis of MC, the indication of primary foci and the monitoring of the disease, and the large-scale single-center data provide a reliable support for its clinical application.

## 1 Introduction

Meningeal carcinomatosis (MC) is a disease caused by the metastasis of moderately advanced malignant cells to the soft cerebrospinal membrane and arachnoid membrane via peripheral blood or cerebrospinal fluid (CSF) (1). Studies have shown that 1% to 5% of patients with solid tumors may develop MC, the incidence of which is closely related to the type of tumor (2), and the prevalence of MC is about 8% of all cancer patients (3). Several autopsy studies have found that the prevalence of MC can be as high as 20% in patients with solid tumors, a figure that suggests that the disease is often misdiagnosed or underdiagnosed, and that its incidence has increased significantly in recent years (4, 5).The clinical presentation of MC usually lacks specificity, and imaging studies often show atypical or ambiguous presentations due to the lack of substantial intracranial lesions. Therefore, early recognition and timely diagnosis of MC have become a major challenge in clinical practice.

CSF cytomorphometry is recognized as the gold standard for MC diagnosis. However, its popularization and application are limited due to the high technical threshold and insufficient knowledge of clinical application, and there are certain limitations. In addition, CSF tumor markers (TM) have outstanding performance in terms of sensitivity and specificity, but the relevant research reports are still limited, and they are not widely used in routine examinations.As an auxiliary test, CSF biochemical indexes can effectively assist in the identification and diagnosis of a variety of intracranial disorders, but their specificity is insufficient, and further research is needed to assess their clinical value. Currently, most descriptive studies of MC are small samples, lacking the integration of multiple indicators for analysis, especially the comparison data between CSF and serum TM are insufficient (6, 7). This study retrospectively analyzed multiple indicators of 209 MC patients diagnosed and treated at Guangdong Sanjiu Brain Hospital from 2021 to 2023. Through the analysis of large samples with multiple indicators, we can fill the research gap of the association between clinical features of MC and CSF indicators, and provide data support for the optimization of the diagnostic process.

## 2 Objects and methods

### 2.1 Objects

**Figure 1:**
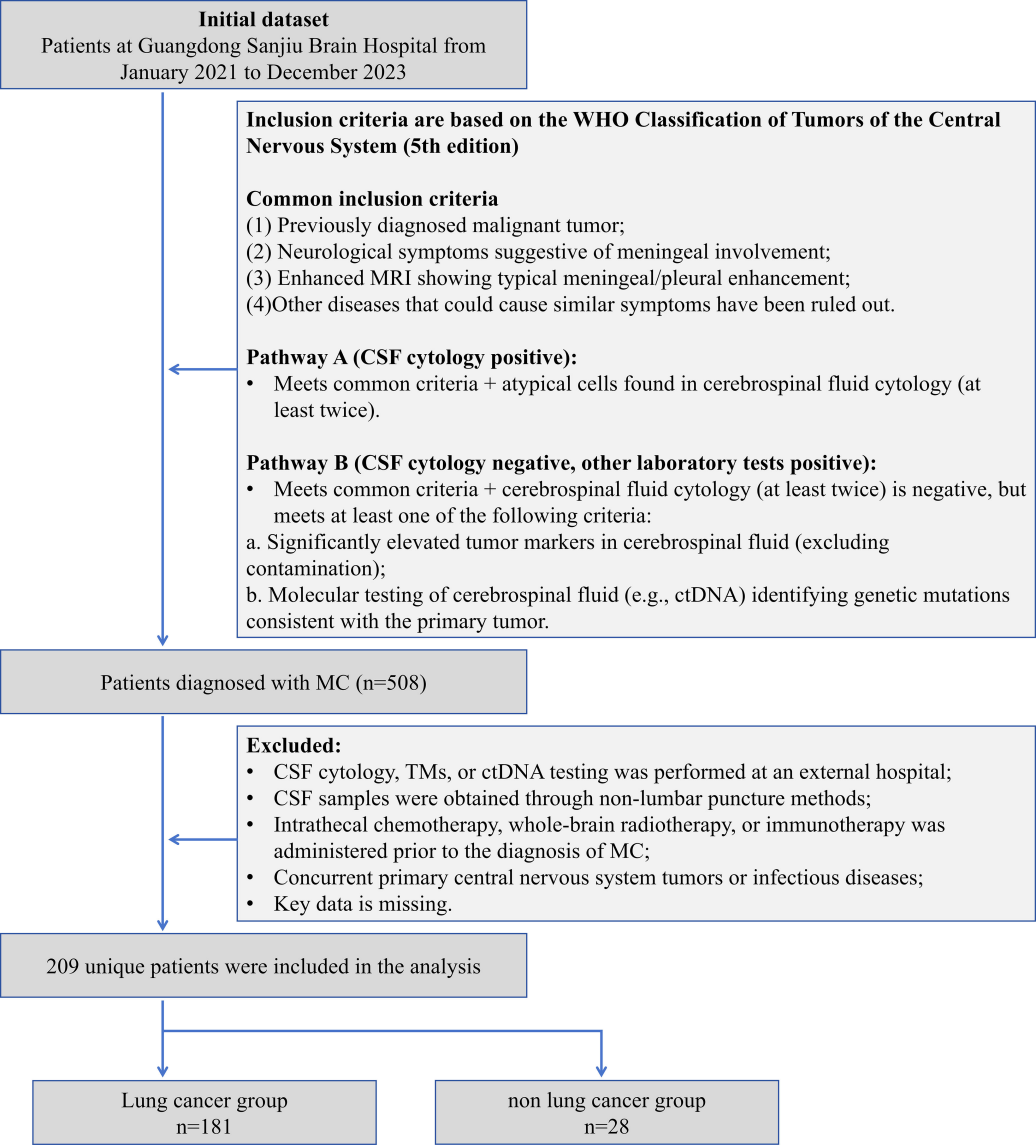
Analysis plan.

This study retrospectively included 209 patients with MC diagnosed from January 2021 to December 2023 at Guangdong Sanjiu Brain Hospital (a public tertiary-level A-class brain specialty hospital). The inclusion criteria were based on the WHO Classification of Tumors of the Central Nervous System (5th edition) (8), and the following conditions had to be fulfilled simultaneously: (1) the presence of a history of malignancy; (2) new-onset neurological symptoms or signs (e.g., headache, cranial nerve palsy); (3) enhancement of MRI showing typical pilonidal enhancement, and exclusion of other disorders such as infection and inflammation; (4) Laboratory confirmation pathway (either of the following can be satisfied): A) more than 2 times of CSF cytology detected atypical cells; B) CSF cytology is negative but meets the following criteria: (i) CSF TM is significantly higher than the serum level (>2 times the upper limit of the reference value) and excludes blood contamination; or (ii) CSF ctDNA detects the driver mutation consistent with the primary tumor. And (5) Completeness of data: The first MC diagnosis was made in our hospital with complete clinical, laboratory and follow-up data. Exclusion criteria were as follows: (1) Key laboratory tests were performed at an outside institution; (2) CSF was obtained by a non-lumbar puncture route, such as ventricular drainage; (3) Patients with MC were treated with intrathecal chemotherapy, radiotherapy, or immunotherapy prior to the diagnosis; (4) Combination of primary central nervous system tumors or active infections; and (5) missing data >20%. Lung cancer is of great clinical significance as one of the malignant tumors with the highest morbidity and mortality rates worldwide. Also, the number of patients with lung cancer sources dominated this study, and their sample size was sufficient to support independent statistical analysis. Meanwhile, the number of MC patients with primary lung cancer dominated this study, and its sample size was sufficient to support independent statistical analysis. Therefore, we categorized MC patients with primary lung cancer into the lung cancer group and MC patients with primary other cancers into the non-lung cancer group, which can further accurately assess the performance of each of the indicators in lung cancer, the most common and clinically significant tumor type, and enhance the statistical validity and clinical generalizability of the study results (**Figure 1**). The study was approved by the Ethics Committee of our hospital (C392024007) and informed consent was waived for retrospective study. Our hospital is a public, tertiary care, specialized brain hospital. As a center for neurological diseases in South China, the hospital’s specialty reputation attracts patients from all over the country, which guarantees the representativeness of the study sample and the reference value for clinical practice.

### 2.2 Experimental methods

An electrochemiluminescence immunoassay analyzer (Cobas e801, Roche, Switzerland) and accompanying TM test reagents, and a fully automated biochemistry analyzer (Cobas c702, Roche, Switzerland) and accompanying biochemistry test reagents were used in this study. All tests were performed using original quality control products, and quality control was performed before each day’s test to ensure that the quality control results complied with the laboratory’s Westgard Multi-Rule before the specimen test was carried out.

3~5 mL of morning venous blood of the enrolled patients was collected, centrifuged for 10 min (2200×g), and the serum was collected for TM test; CSF of the enrolled patients was taken by lumbar puncture, and was sent to be examined with a sterile tube that was matched in the lumbar puncture kit. The volume of specimen for CSF cytology is not less than 2 ml, and the volume of specimen for CSF biochemical indexes and tumor markers is about 2 ml respectively. CSF specimens were centrifuged at 4 °C for 15 minutes (2200 × g) after collection and tested immediately. Serum and CSF supernatants used in the study were dispensed and stored at -80 °C.

TM indices contained Alpha-Fetoprotein (AFP), Cancer Antigen 125 (CA125), Carcinoembryonic Antigen (CEA), Cytokeratin 19 Fragment (Cyfra21-1), and Squamous Epithelial Cell Carcinoma Antigen (SCC). TM assays for CSF specimens were consistent with serum. While the biochemical indices contain Adenosine Deaminase (ADA), Aspartate Aminotransferase (AST), Chloride Ion (Cl^-^), Protein, Glucose (GLU), Lactic Acid (LAC) and Lactate Dehydrogenase (LDH). The reference intervals were determined according to specifications, literature (9) and laboratory settings. The specific reference intervals, the lowest limit of detection and methodological validation parameters are listed in **Supplementary Table S1** of the Supplementary Material.

In order to perform testing of CSF cytology, CSF specimens were required to be sent for testing in a timely manner. Cell collection was performed using a cell smear centrifuge, centrifuged at 68×g for 10 min, and the active components were enriched on slides. The slides were dried and stained with Rachel-Giemsa stain, and finally read and reported by two experienced laboratory technicians.

The morphological characteristics of atypical cells include: (1) Large cell size, enlarged nucleus, prominent nucleolus, and an increased nuclear-to-cytoplasmic (N:C) ratio; (2) Tendency to form clusters with indistinct cell borders; (3) Intense cytoplasmic basophilia, manifesting as blue or deep blue staining of the cytoplasm; (4) Pleomorphism (variation in shape) and anisocytosis (variation in size); (5) Presence of single or multiple nuclei, with nuclear pleomorphism; (6) Nucleoli that may be single, multiple, or inconspicuous; (7) Nuclear chromatin that is coarse or fine; (8) Irregular cell borders, which may show knobby or pseudopod-like projections; (9) Cytoplasm that may exhibit prominent vacuoles or specific pigment granules; (10) Cell membranes that may show knobby or pseudopod-like projections. Atypical cells originating from different sources possess distinctive morphological features.

### 2.3 Statistical methods

Data analysis was conducted using SPSS Statistics version 21.0. Continuous variables were initially assessed for normality via the Shapiro-Wilk test. Those conforming to normal distribution were expressed as mean ± standard deviation (SD) and compared between groups using the independent samples t-test (when variances were homogeneous) or Welch’s t-test (when variances were heterogeneous). Non-normally distributed variables were reported as median with interquartile range [M (Q1, Q3)], with group comparisons performed using the Mann-Whitney U test. For paired comparisons of identical indicators across different specimen types, normally distributed data employed the paired t-test while non-normal data utilized the Wilcoxon signed-rank test. Categorical data were presented as frequency (percentage) [n (%)] and analyzed with the chi-square test. Statistical significance was defined as a two-tailed P-value < 0.05.

## 3 Results

### 3.1 General information

This study enrolled 209 patients with MC. The primary malignancies were predominantly lung cancer (n=181, 86.60%), followed by breast cancer (n=16, 7.66%), melanoma (n=5, 2.39%), gastric cancer (n=4, 1.91%), with single cases (0.48% each) of cervical cancer, rhabdomyosarcoma, and maxillary sinus carcinoma. The principal clinical manifestation involved leptomeningeal involvement of the cerebral hemispheres (n=198, 94.74%), with frequent symptoms including headache (n=137, 65.55%), dizziness (n=73, 34.93%), and altered mental status (n=46, 22.01%). Additionally, some patients exhibited blurred vision (n=62, 29.67%), limb weakness (n=59, 28.23%), and cognitive impairment (n=11, 5.26%) (**Table 1**).

**Table 1:** Clinical characteristics.

Category	MC N(%)	Lung cancer group N(%)	Non-lung cancer group N(%)	*X^2^*/t	*P*
Gender (Male/Female)	98/111	87/94	11/17	0.808	0.369
Age (Range, yr)	54.44(20-77)	54.86(27-76)	51.71(20-77)	1.237	0.218
Smoking history					
Previous or current smoking	72(34.45)	67(37.02)	5(17.86)	3.858	0.051
No smoking	137(65.55)	114(62.98)	23(82.14)		
Symptoms					
Headache	137(65.55)	121(66.85)	16(57.14)	0.992	0.319
Dizziness	73(34.93)	66(36.46)	7(25.00)	1.431	0.232
Consciousness	46(22.01)	38(20.99)	8(28.57)	0.795	0.373
Blurred vision	62(29.67)	50(27.62)	12(42.86)	2.660	0.103
Limb weakness	59(28.23)	52(28.73)	7(25.00)	0.161	0.688
Cognitive impairment	11(5.26)	8(4.42)	3(10.71)	1.906	0.167
MRI abnormalities					
Scan without contrast	26(12.44)	19(10.50)	7(25.00)	4.701	0.030
Scan with contrast	125(59.80)	107(59.12)	18(64.29)	0.423	0.810

### 3.2 Imaging features

The enrolled patients had a mean age of 54.44 years (range: 20-77) with no significant intergroup differences in sex distribution (P = 0.369). Smoking history was more prevalent in the lung cancer subgroup (37.02% vs 17.86%, P = 0.062). Headache (65.55%), dizziness (34.93%), and altered mental status (22.01%) were the most common symptoms, demonstrating no statistically significant differences in distribution across groups (all P>0.05). All patients underwent cranial imaging (CT and MRI) upon admission. Non-contrast scans showed diagnostic irrelevance to MC in 183 cases (87.56%), with observed abnormalities primarily manifesting as cerebral edema, multiple ischemic foci, and non-specific white matter changes. Among 186 patients (88.52%) who received contrast-enhanced cranial MRI, 125 (67.20%) exhibited leptomeningeal enhancement presenting as irregular linear, nodular, or nodular-like patterns (**Table 1**).

### 3.3 TM levels in serum and cerebrospinal fluid

Comparisons between lung cancer and non-lung cancer groups revealed statistically significant differences in CEA levels both in CSF (P = 0.020) and serum (P = 0.006). CYFRA21–1 showed intergroup differences only in CSF (P = 0.043), whereas AFP, CA125, and SCC demonstrated no significant differences in either specimen type (all P>0.05).

Analyses across specimen types indicated statistically significant differences between CSF and serum levels for all markers: AFP, CA125, CEA, CYFRA21-1, and SCC. Specifically, AFP, CA125, and CYFRA21–1 exhibited highly significant differences (P<0.001) (**Table 2**).

**Table 2:** Comparison of tumor marker levels in cerebrospinal fluid and serum between lung cancer and non-lung cancer groups with MC.

Marker (Unit)	Specimen	Lung cancer group M (P25, P75)	Non-lung cancer group M (P25, P75)	Group comparison		Cerebrospinal fluid (CSF) vs Serum	
				Z	*P*	Z	*P*
AFP (ng/ml)	CSF	0.99 (0.91, 1.01)	0.91 (0.91, 1.02)	0.477	0.727	-2.521	<0.001
	Serum	3.57 (2.73, 4.27)	3.24 (2.15, 4.06)	0.779	0.781		
CA125 (U/ml)	CSF	1.08 (0.60, 26.50)	1.13 (0.72, 23.71)	0.482	0.513	-4.610	<0.001
	Serum	24.01 (11.70, 53.22)	20.71 (11.85, 52.55)	-0.239	0.811		
CEA (ng/ml)	CSF	29.70 (2.95, 120.25)	2.51 (0.33, 84.38)	-2.319	0.020	-3.211	0.015
	Serum	19.70 (4.66, 102.50)	3.11 (0.98, 7.46)	-2.739	0.006		
CYFEA21-1 (ng/ml)	CSF	7.65 (2.79, 16.50)	5.68 (1.40, 12.33)	2.021	0.043	6.516	<0.001
	Serum	5.49 (1.68, 7.05)	4.65 (1.19, 5.20)	0.571	0.673		
HCG (IU/L)	CSF	0.83 (0.21, 1.71)	0.73 (0.11, 1.34)	0.987	0.323	-2.468	0.021
	Serum	1.40 (0.85, 2.22)	1.27 (0.55, 2.79)	0.271	0.723		
SCC (ng/ml)	CSF	0.99 (0.91, 1.01)	0.91 (0.91, 1.02)	0.477	0.727	-2.521	<0.001
	Serum	3.57 (2.73, 4.27)	3.24 (2.15, 4.06)	0.779	0.781		

### 3.4 Levels of cerebrospinal fluid biochemical indicators

Biochemical analysis of CSF revealed abnormalities in 201 MC patients (96.17%). Significant differences between lung cancer and non-lung cancer groups were observed in total protein (Pro, P = 0.011) and glucose (GLU, P = 0.036) levels. However, no significant intergroup differences were detected for ADA, AST, CL⁻, LAC, or LDH (all P>0.05). Concentrations of LDH, total protein, and LAC were significantly elevated above the upper reference limits, while glucose levels were markedly reduced below the lower reference limit. ADA, AST, and Cl⁻ levels remained predominantly within normal reference intervals (**Table 3**).

**Table 3:** Comparison of cerebrospinal fluid biochemical markers between lung cancer and non-lung cancer groups.

Biomarker (Unit)	Total MC cohort M (P25, P75)	Lung cancer group M (P25, P75)	Non-lung cancer group M (P25, P75)	Z	P
ADA (U/L)	0.90 (0.50, 1.40)	0.90 (0.55, 1.40)	1.00 (0.50, 1.48)	-0.050	0.961
AST (U/L)	17.10 (12.75, 22.05)	17.30 (13.25, 22.20)	16.30 (8.40, 21.43)	-1.864	0.622
CL- (mmol/L)	121.70 (117.75, 124.55)	121.50 (117.75, 124.60)	123.60 (116.48, 124.45)	0.453	0.650
Pro (g/L)	0.61 (0.34, 1.05)	0.65 (0.37, 1.09)	0.37 (0.12, 0.82)	-2.554	0.011
GLU (mmol/L)	3.20 (2.20, 3.20)	3.10 (2.20, 3.80)	3.70 (2.50, 4.60)	2.096	0.036
LAC (mmol/L)	2.80 (1.98, 4.23)	2.81 (1.96, 4.19)	2.70 (2.05, 4.84)	0.361	0.718
LDH (U/L)	42.00 (28.25, 77.55)	42.10 (29.45, 78.20)	33.65 (14.48,72.30)	-1.563	0.118

### 3.5 Cerebrospinal fluid cytology results

**Figure 2:**
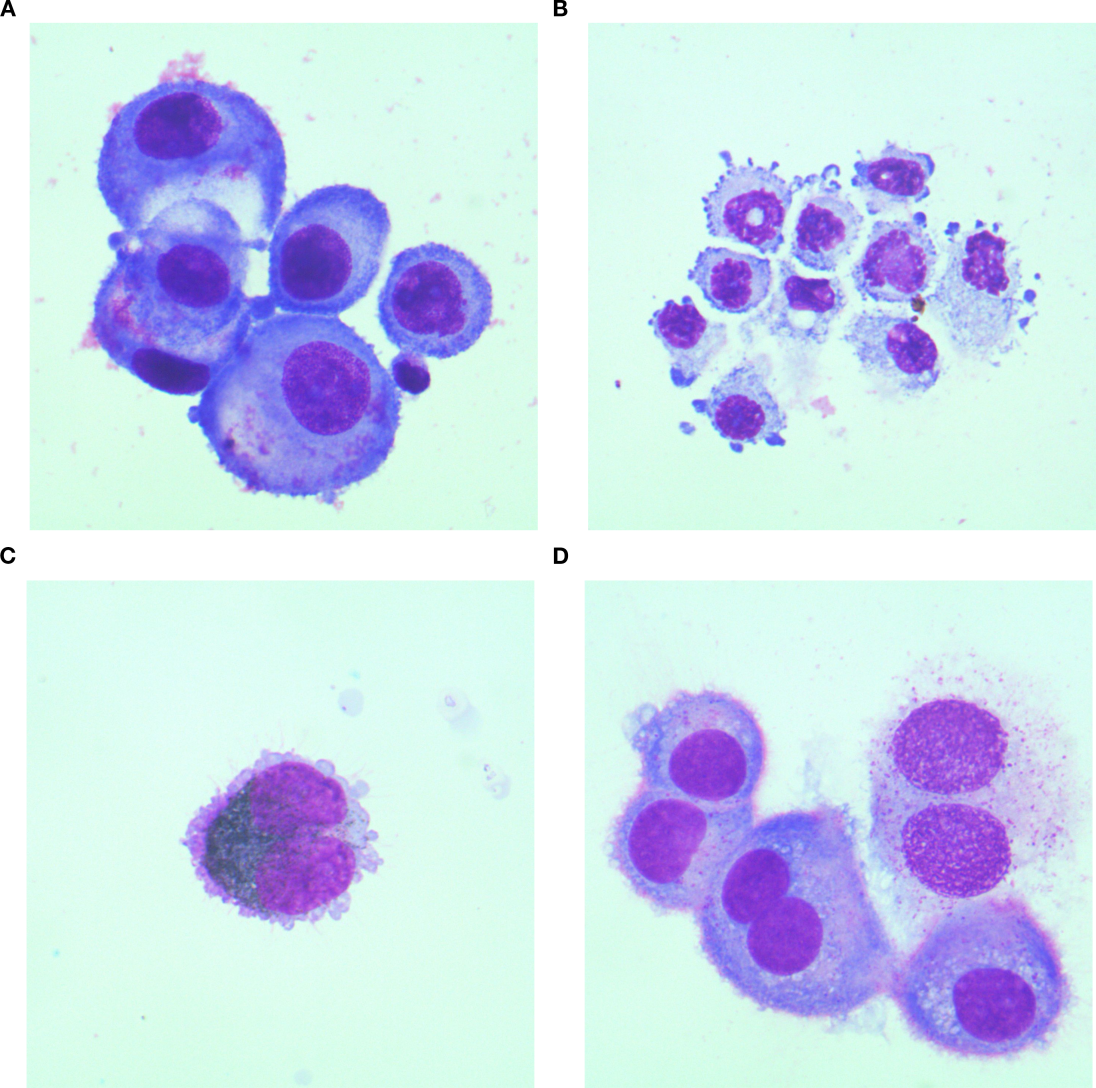
Cytomorphological features of CSF in MC patients originating from different primary malignancies. **(A)** Lung cancer specimen: Atypical cells exhibit marked size variation, with centrally or eccentrically located nuclei and abundant cytoplasm. Vacuolated cytoplasm is observed in a subset of cells; **(B)** Gastric cancer specimen: Atypical cells display pleomorphic nuclei, faintly stained cytoplasm, and tumor-like budding protrusions on the cell membrane; **(C)** Melanoma specimen: Atypical cells containing abundant cytoplasmic melanin granules are identified; **(D)** Breast cancer specimen:Atypical cells present as uninucleated or binucleated forms with moderately abundant cytoplasm, wherein metachromatic granules are consistently observed. All cytological preparations were first Wright-Giemsa stained and subsequently examined under 1000× magnification.

**Figure 3:**
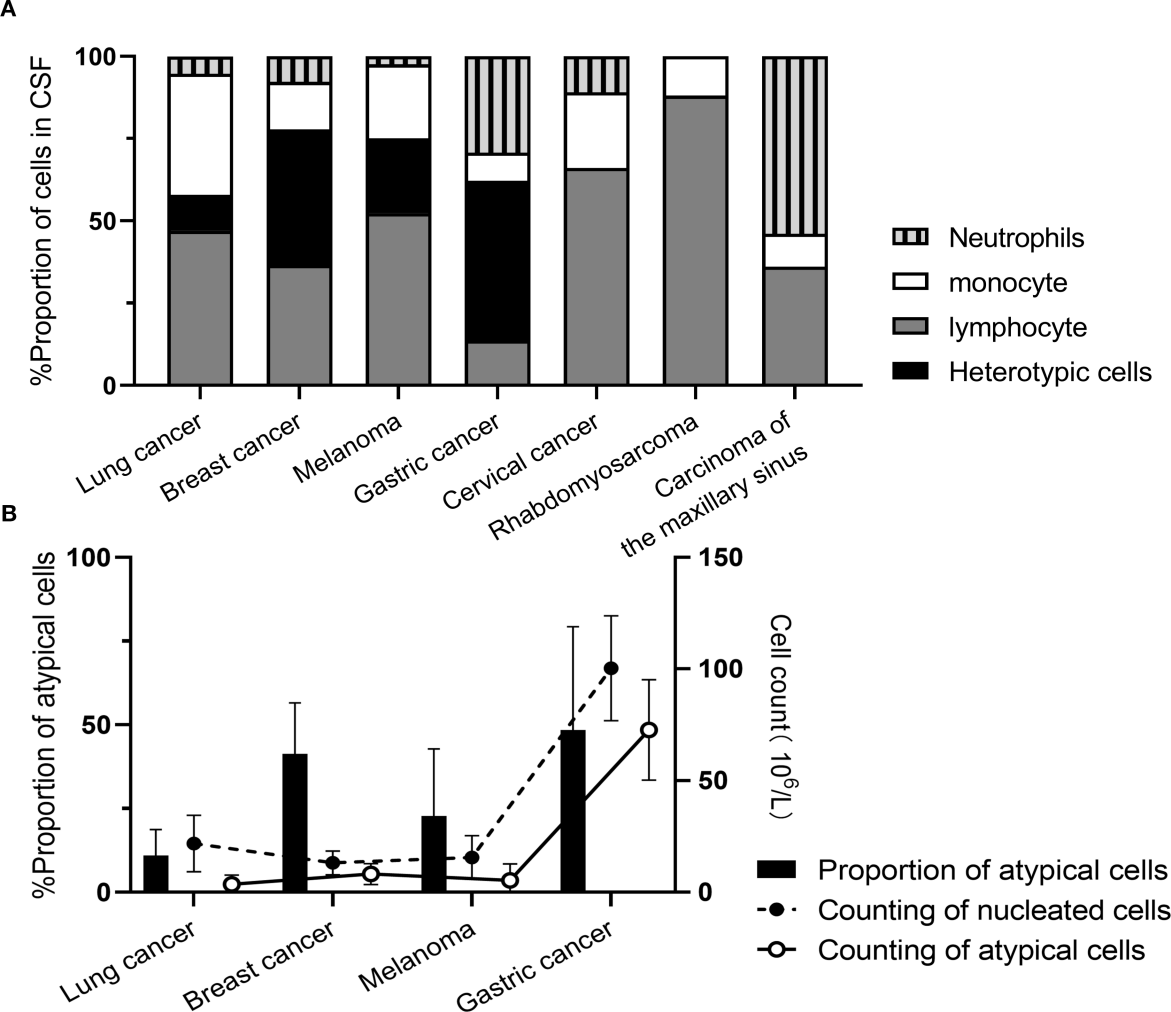
Cytological enumeration results in MC patients originating from different primary malignancies. **(A)** Proportion of nucleated cells; **(B)** Enumeration of nucleated cells.

During initial CSF cytological examination, atypical cells were detected in 199 patients (95.22%), with morphology varying by primary tumor origin (**Figure 2**). Two patients (0.95%) exhibited atypical cells on repeat testing (second examination), and one (0.48%) at the sixth examination. Four patients (1.91%) had negative initial results without subsequent testing. Three patients (1.44%) showed no atypical cells despite >6 CSF cytological examinations. CSF specimens appeared: colorless in 158 cases (75.60%), straw/yellow in 39 (18.66%), pinkish/red in 10 (4.78%), and opalescent in 2 (0.96%). Clarity was observed in 193 samples (92.34%), with 16 (7.66%) showing mild turbidity or opacification. Abnormal globulin levels were identified by Pandy’s test in 101 cases (48.36%). Elevated RBC counts (>2×10^6^/mL) occurred in 124 patients (59.33%), and nucleated cell counts (>5×10^6^/mL) in 122 (58.37%). The proportion of atypical cells in CSF was: lung cancer-derived MC: 4% (0%, 10%); breast cancer: 3.5% (0%, 54.5%); melanoma: 3.0% (0%, 44.0%); gastric cancer: 54.0% (16.5%, 73.5%) (**Figure 3**).

### 3.6 Dynamic observation of cerebrospinal fluid indicators

**Figure 4:**
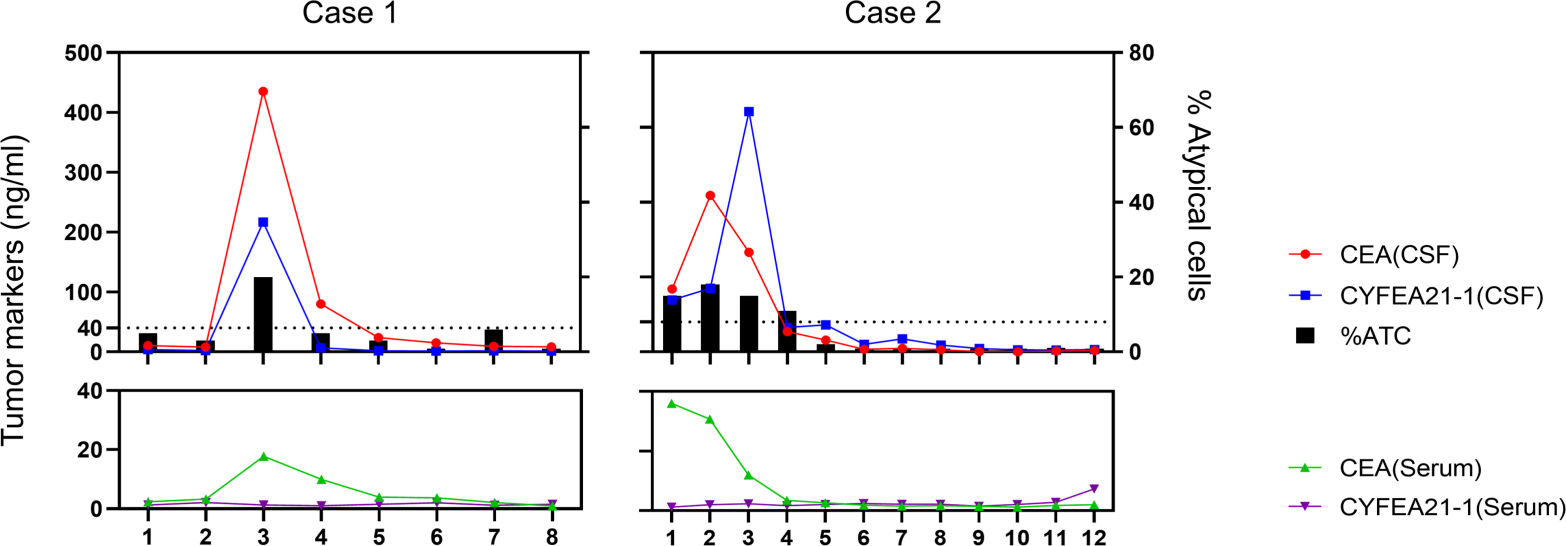
Serial monitoring of cerebrospinal fluid and serum parameters in two patients undergoing intrathecal chemotherapy.

Representative findings are depicted in **Figure 4**. Two patients showed differential proportions of atypical cells in CSF during initial examination, yet both were definitively diagnosed with MC when integrated with primary malignancy identification. Patient 1 underwent eight serial CSF analyses throughout the clinical course. The third assessment (post-discharge surveillance) revealed dramatically elevated tumor marker levels in CSF compared to serum, with escalating trends paralleling increased proportions of atypical cells – indicative of intracranial relapse. Subsequent therapeutic intervention achieved effective control of both tumor marker levels and atypical cell proportions. Patient 2 presented with significantly elevated CSF tumor markers at initial diagnosis. Following treatment, tumor markers normalized concomitant with progressive decline in atypical cell proportions culminating in complete clearance, correlating with amelioration of clinical symptoms.

## 4 Discussion

Meningeal carcinomatosis manifests with insidious onset and non-specific clinical features, frequently leading to delayed diagnosis that forfeits optimal therapeutic windows. As evidenced by (10), MC may complicate virtually all malignancies, with lung cancer (25%), melanoma (23%), and breast cancer (5%) constituting the most prevalent primary origins—findings largely congruent with our study, indicating global consistency in primary tumor spectra. Notably, the proportional distribution exhibits geographical variation: Our cohort demonstrated significantly higher lung cancer-derived MC incidence (86.60%), potentially attributable to China’s elevated lung cancer burden (11) and the widespread therapeutic implementation of epidermal growth factor receptor tyrosine kinase inhibitors (EGFR-TKIs) (12). This study’s expanded sample size enhances the precision of real-world epidemiological representation. Concurrently, extant research (13) confirms lung cancer’s exceptional metastatic propensity, with ≈57% of patients presenting with metastases at initial diagnosis, frequently involving the central nervous system (CNS) (14). Consequently, vigilant surveillance for early detection and prevention of neoplastic dissemination is warranted in cancer patients, particularly those with pulmonary primaries.

Malignant cells disseminate to the CNS via hematogenous spread, perineural/intraneural routes, or direct extension from cerebral/calvarial metastases (15), subsequently infiltrating the CSF; circulating malignant cells colonize diverse neuroanatomical sites through CSF flow, conferring the multifocal and polymorphic neurological manifestations characteristic of MC—exemplified by meningeal irritation upon subarachnoid metastasis versus radicular pain and motor deficits from spinal nerve root involvement. The most prevalent symptoms (headache, nausea, vomiting) and progressive manifestations (cranial neuropathies, cognitive impairment, psychiatric disturbances, personality alterations, seizures, diplopia, external ophthalmoplegia) (16) align with our data. This non-specific symptomatology combined with the absence of definitive biomarkers underlies frequent misdiagnosis, posing substantial clinical diagnostic challenges.

The limited sensitivity of neuroimaging examinations poses additional diagnostic challenges for early MC detection. Magnetic resonance imaging, demonstrating superior capability in visualizing diverse patterns of cranial nerve enhancement and nodular abnormalities compared to CT, is established as the preferred imaging modality for MC diagnosis (17). As documented (18), characteristic leptomeningeal enhancement typically manifests as diffuse thickened enhancement with focal nodular components, whereas unenhanced MRI frequently fails to detect meningeal pathology—a phenomenon potentially attributable to neoplastic infiltration patterns. Following CNS dissemination, malignant cells diffusely distribute along the leptomeninges, attenuating density differentials between pathological and adjacent normal tissues, necessitating contrast-enhanced MRI for effective MC diagnostic support (19). Notably, enhanced MRI exhibits persistent false-negative rates up to 30% (20), aligning with our findings.

Compared to neuroimaging and symptom-based diagnostic approaches, CSF examination demonstrates superior sensitivity and specificity. CSF analysis typically includes routine, biochemical, and cytological testing, with cytology recognized as the gold standard for MC diagnosis due to its exceptionally high specificity, though sensitivity remains relatively low. The initial lumbar puncture yields approximately 50% sensitivity, while repeated sampling significantly enhances detection rates to 80%. In this study, MC patients exhibited 95.22% positivity at first examination and 96.65% after repeated sampling, exceeding literature reports. Notably, as the proportion of atypical cells does not proportionally correlate with total cell counts, cytology retains diagnostic value even in cases with extremely low cellularity or minimal atypical cell ratios. Technological advancements in equipment, improved slide preparation techniques, and accumulated operator experience have collectively enhanced cytological detection efficacy, necessitating increased investment in specialized cytopathology training (24). Nevertheless, persistent false-negative cases occurred despite multiple examinations, ultimately requiring alternative methods for MC confirmation—paralleling contrast-enhanced MRI’s ≈30% false-negative rate. This limitation arises from two distinct neoplastic growth patterns in the leptomeninges (21): (1) adherent flat spreading and (2) floating clustered proliferation. Cytology detects most floating-phenotype cells and only those adherent-type cells exfoliated into CSF, constituting an inherent diagnostic constraint. Subgroup analysis revealed gastric cancer-derived MC patients demonstrated higher atypical cell proportions and altered neutrophil/monocyte/lymphocyte ratios, indicating tumor-specific variations in immune responses. Therefore, integrating ancillary laboratory parameters for comprehensive patient assessment is essential to support diagnosis and treatment planning (22). In our cohort, MC patients showed marked qualitative CSF protein abnormalities consistent with biochemical results, concurrently exhibiting significantly elevated LAC and LDH levels—attributable to impaired oxygen utilization and anaerobic glucose metabolism (23, 24). Lung cancer-derived MC cases manifested disproportionately higher CSF protein and lower glucose levels, likely reflecting more aggressive blood-brain barrier disruption that exacerbates protein leakage and accelerates glucose consumption.

Consequently, TMs in CSF demonstrate significant utility for early auxiliary diagnosis of MC (9, 25), with our data revealing markedly elevated CEA levels in both serum and CSF exceeding reference intervals—particularly higher in lung cancer-derived MC patients. These findings indicate tumor-origin-specific TM monitoring strategies for optimized diagnosis. Although TM elevations occur in serum and CSF, concentration gradients may substantially differ, primarily attributable to two mechanisms (26): trans-blood-brain barrier (BBB) diffusion post-disruption versus direct intrathecal release by atypical cells. Notably, CYFRA 21–1 concentrations in CSF significantly surpassed serum levels in MC patients, explained through multifactorial pathways: Local intrathecal synthesis constitutes the core mechanism, whereby atypical cells metastasized to leptomeninges upregulate cytokeratin 19 (CK-19) expression via clonal selection or CNS microenvironmental induction—CK-19 overexpression being mechanistically linked to aggressive metastasis—directly releasing CYFRA21–1 into CSF (27). Concurrently, BBB equilibrium dynamics play critical roles: despite CYFRA21-1’s lower molecular weight conferring greater theoretical BBB permeability than CEA, intrathecal tumor output overwhelms BBB clearance capacity (28, 29). Furthermore, differential clearance kinetics amplify the gradient: CYFRA21-1’s serum half-life is substantially shorter than CEA’s, limiting plasma accumulation, whereas CSF clearance relies predominantly on BBB transit lagging behind local secretion rates (30). These mechanisms collectively enhance CSF CYFRA21-1’s diagnostic sensitivity/specificity for leptomeningeal metastasis, warranting CSF TM analysis even with normal serum levels—especially given literature documentation (31) of CSF TM elevations often preceding MRI abnormalities. Thus, any TM aberration provides critical diagnostic clues for suspected MC, with integrated CSF cytology and TM profiling facilitating early detection.

Liquid biopsy technologies, exemplified by CSF circulating tumor DNA (ctDNA) analysis, demonstrate transformative potential in diagnosing and managing meningeal carcinomatosis, offering novel perspectives to overcome traditional limitations (32, 33). CSF ctDNA precisely mirrors central nervous system tumor genomic profiles, enhancing diagnostic sensitivity while enabling real-time therapeutic monitoring through dynamic mutation burden tracking—thereby guiding targeted therapy adjustments (34). Applications of high-throughput sequencing (NGS) and low-pass whole-genome sequencing (LP-WGS) further extend its utility in multi-gene variant detection and personalized surveillance, particularly showing unique advantages in pediatric brain tumors (6, 35). Nevertheless, widespread implementation faces practical challenges: standardization deficits in sensitivity (especially for low tumor burden), elevated costs, and dependency on sophisticated equipment/specialized teams, limiting accessibility in primary care facilities. Against this backdrop, conventional diagnostic methods retain indispensable foundational roles. Future precision management will likely integrate ctDNA with cytology, biochemical assays, and TMs—scaffolding the framework with traditional techniques while refining details through molecular profiling—to collaboratively construct a macro-micro integrated diagnostic system that optimizes clinical decision-making and patient outcomes.

This study has several limitations: the substantial predominance of lung cancer-derived MC cases with underrepresentation of other primary origins may bias diagnostic metric performance toward lung cancer characteristics, necessitating caution when extrapolating findings to other MC subtypes and mandating future studies with balanced cohorts to clarify cross-type variations; its cross-sectional design captures only baseline parameters at diagnosis, lacking longitudinal monitoring during therapy and long-term prognostic data, thereby precluding establishment of a comprehensive “biomarker-treatment-outcome” evidence chain; concurrently, the single-center retrospective nature introduces potential selection bias through institutional protocols that may miss atypical-symptom MC patients or exhibit heightened lumbar puncture utilization for specific primaries, further compromising cohort equilibrium. These constraints highlight avenues for future research—enhancing diagnostic and management evidence through expanded sample diversity, prospective designs, and integrated long-term follow-up.

## 5 Conclusion

This study strongly supports the comprehensive strategy of combining CSF biochemical indicators, tumor markers and cytology. This strategy has important clinical application value for the early diagnosis of MC, primary lesion indication and dynamic monitoring of the disease. Its large-scale single-center data provides a reliable evidence-based basis for the clinical diagnosis and treatment of MC.

## Data Availability

The original contributions presented in the study are included in the article/**Supplementary Material**. Further inquiries can be directed to the corresponding author.

## Supplementary material

Supplementary Table 1 Summary of performance validation for serum tumor markers and cerebrospinal fluid testing projects.
